# The Late Appearance of Agglutinins in Paratyphoid a Fever

**Published:** 1919

**Authors:** 


**The Late Appearance of Agglutinins in Paratyphoid A Fever.** By Capt. W. M. MacAdam, R. A. M. C. *Journal of the Royal Army Medical Corps*, September 1918.

The author gives a series of observations made on 33 acute cases, mostly paratyphoid A fevers, which may be summarized as follows :

*Table Showing the Date of Appearance of the Highest Para A Titre.*

| | Series inoculated with T. A. B. | Series inoculated with T. V. |
|---|---|---|
| (1) First 3 weeks . . . . . . . . . . | Nil | Nil |
| (2) 4th and 5th weeks . . . . . . . . | 6 | 5 |
| (3) 6th " 7th " . . . . . . . . | 8 | 5 |
| (4) 8th week and onwards . . . . . . | 5 | 4 |

From 14 of these cases, the infective organism has been isolated either by means of blood culture or from the feces. The agglutination reactions have been carried out at intervals from the 1st to the 20th week of the fever. As seen from the above table, in a large number of cases the agglutinins for B. Paratyphosus A are very late in their appearance.

*The Extent of Production of Para A Agglutinins.*

The presence of Para A agglutinins may, in some cases, be revealed by agglutination tests carried out in low dilutions of the sera. This, however, is by no means constant, as the agglutinins often appear at a comparatively late period of convalescence. Laboratory tests should not, therefore, be confined to the period of pyrexia or early convalescence, but continued later.

*The Question of the Relative Diminution or Absence of the Inoculation Agglutinins during Pyrexia.*

In two-thirds of the cases observed, in which agglutination tests have been carried out during the first and second weeks of pyrexia, a marked diminution of the inoculation agglutinins has been noted. In cases of malaria and other acute non-enteric febrile conditions, no such diminution of the agglutinins resulting from previous T. V. or T. A. B. inoculation has been detected. No relation has been found either between the occurrence of relapses and an alteration in the agglutination curve, or between the end of the pyrexia and the date of record of the maximum agglutinin content.

*The Agglutination Curve of Bacillus Typhosus in Paratyphoid Infections.*

A marked ascent in the B. Typhosus agglutination usually precedes or is synchronous with the appearance or increased production of Para A agglutinins; this increase shows the futility of basing the statistics on the relative incidence of fevers diagnosed from tests carried out during pyrexia or early convalescence in cases where blood cultures have either proved negative or been neglected.

The value of agglutination reaction would be considerable in the determination of the relationship between inoculation and infection agglutinins, and in the study of the processes on which the agglutination phenomenon depends, if the agglutination curve were established as the result of a series of tests repeated at short intervals and continued well into convalescence.

